# Ranula: Modified Micro-Marsupialization: Case Report and Review of Literature

**DOI:** 10.22038/IJORL.2023.61890.3131

**Published:** 2023-03

**Authors:** Barbara Verro, Rodolfo Mauceri, Giuseppina Campisi, Carmelo Saraniti

**Affiliations:** 1 *Department of Biomedicine, Neuroscience and Advanced Diagnostic, University of Palermo, Palermo, Italy.*; 2 *Department of Surgical, Oncological and Oral Sciences (DiChirOnS), University of Palermo, Palermo, Italy.*

**Keywords:** Oral surgery, Oral pathology, Ranula, Ranula surgery, Salivary gland disease

## Abstract

**Introduction:**

Ranula is a limited mucus retention on the floor of mouth. Due to the young age of patients, over the years, attempts were made to find minimally invasive and effective surgical techniques. To date, however, there is still no gold standard. The modified micro-marsupialization is an effective and minimally invasive technique, with minimal risk of relapse, although there are very few reports about it.

**Case Report:**

A 12-year-old male presented to our ENT Clinic with a rounded swelling with regular and defined margins, measuring 4x3 cm, soft and painless, non-compressible and bluish. Clinical diagnosis of ranula was made and a modified micro-marsupialization was performed: eight interrupted sutures using silk 3-0 were placed perpendicularly to the major axis of the lesion, from one side of the lesion to the other, without reaching the underlying tissue. No sutures were lost during follow-up, no complications occurred. Complete healing was reached after removing sutures on the 30th postoperative day. At 6 months control no relapse was observed.

**Conclusion:**

Modified micro-marsupialization is strongly indicated and recommended, especially in pediatric patient, due to its low invasiveness and its very low relapse rate. The poor case history found in the literature is probably an indication of the lack of knowledge of modified micro-marsupialization which, in our opinion, could be considered the gold standard.

## Introduction

Ranula is a limited mucus retention on the floor of mouth. The word derives from the Latin “rana”, that is frog, because the lesion clinically recalls the frog belly (1,2). This mucus retention is typically related to a trauma of the excretory duct of salivary gland (2). Usually, these lesions occur in the first two decades of life and clinical examination is sufficient to make diagnosis (3), although other investigations such as ultrasound or computed tomography (CT) may be performed (4).

Ranula therapy is surgical. However, the young age of patients is the main reason why, over the years, attempts were made to find minimally invasive and, at the same time, effective surgical techniques. To date, however, there is still no gold standard. The first therapeutic strategies were: 1) sclerotherapy or 2) incision and drainage or 3) ranula excision which, however, present a high risk of recurrence (4,5), 4) ranula and sublingual gland excision with a high risk of complications (lingual nerve injury, Wharton duct injury, bleeding and / or wound dehiscence) (4,6). Therefore, in 1995, Morton et al. proposed a new minimally invasive surgical technique (7), the so-called marsupialization, which consisted in placing a single silk suture at the dome of cyst (7). However, this surgical approach was associated with high rates of recurrence (8). Therefore, in 2000, Delbem et al. (2) have proposed a modification in marsupialization technique, called micro-marsupialization. In this case, a single 4-0 silk suture through the internal part of ranula, along its largest diameter, was placed and removed after 7 days. Although this approach was minimally invasive, it was not effective due to and high rate of ranula recurrence within 30 days. So, in 2007, on the basis of the techniques introduced by Morton and Delbem, a new surgical technique was proposed by Sandrini et al. (9): the modified micro-marsupialization. The modified technique introduced contemplate the following procedures: greater number of stitches, shorter distance between the point of entry and exit of the needle, permanence of the suture for at least 30 days. However, even if this technique is effective and minimally invasive, with minimal risk of relapse, there are very few reports about it (9-13). 

In this study, we report a clinical case of ranula in a child treated with the latest proposed surgical technique and a review of the literature on modified micro-marsupialization.

## Case Report

**Fig 1:**
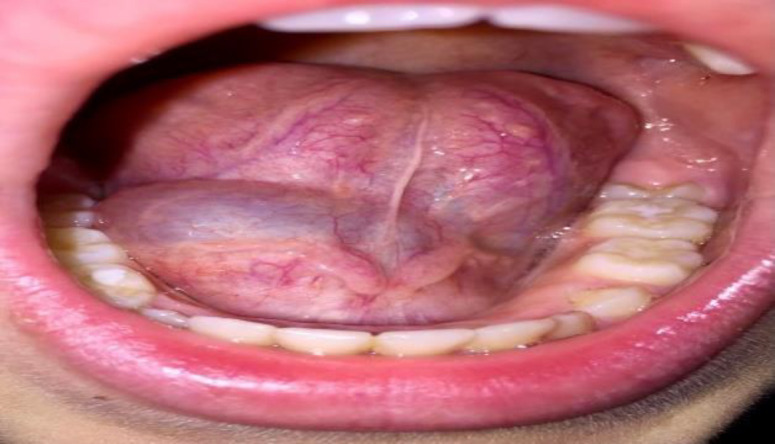
Pre-operative lesion

In 2020, a 12-year-old male presented to our ENT Clinic reporting a swelling on the floor of the mouth on the right since 2 months, without paresthesia, pain, dysphagia. During oropharyngoscopy, a rounded swelling with regular and defined margins, measuring 4x3 cm, soft and painless, non-compressible, bluish was appreciated. The surface of the swelling showed no signs of infection or ulceration. It extended along the right sublingual region (fig.1). Thus, clinical diagnosis of ranula was made and surgery was indicated.

**Fig 2:**
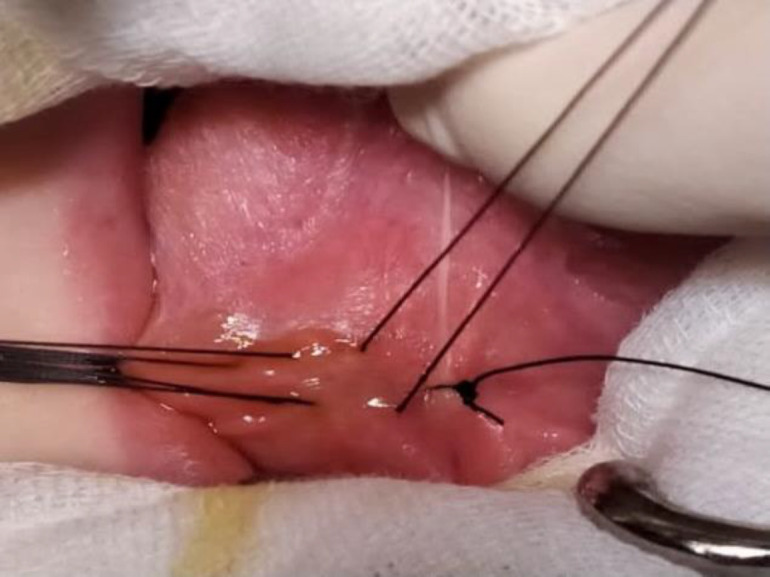
Modified micro-marsupialization technique

Surgical protocol included: 1) disinfection of the area with 0.1% povidone iodine solution and local anesthesia with lidocaine spray were performed; 2) eight interrupted sutures using silk 3-0 were placed perpendicularly to the major axis of the lesion, from one side of the lesion to the other, without reaching the underlying tissue; 3) compression with the swelling finger allowed the mucin to drain through the punctures by stitches; 4) drainage was completed with an incision of 3 mm between two sutures; 5) surgical knots were tied (fig.2).

**Fig 3:**
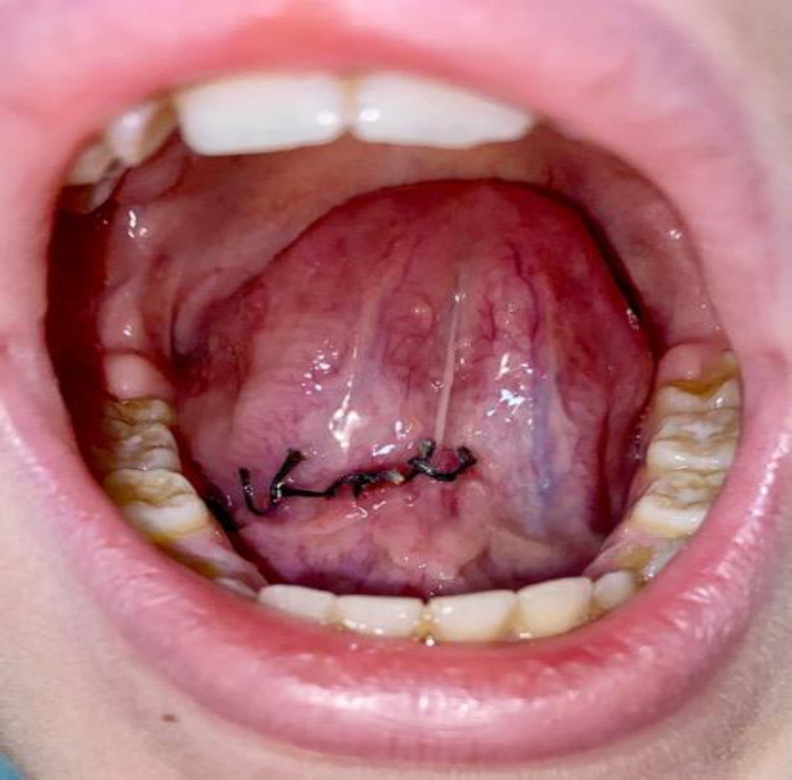
30th postoperative day before sutures removing

Post-operative, the patient was instructed to maintain proper oral hygiene and to use 0.2% chlorhexidine mouthwash 3 times a day to avoid surgical site infections. During the follow-up, weekly controls were made. No sutures were lost during follow-up, no edema or surgical site infections occurred, with uneventful clinical healing (fig.3). 

**Fig 4:**
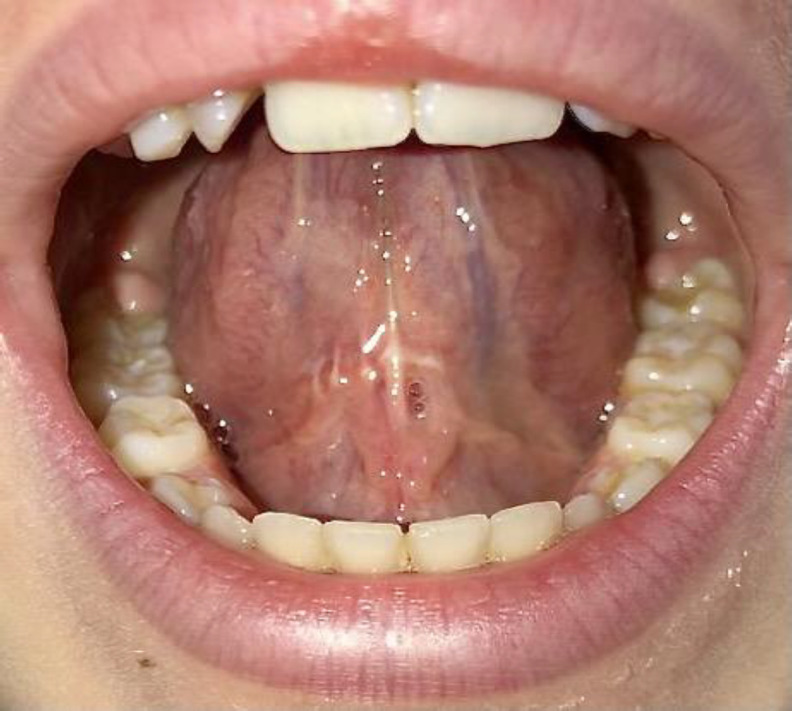
Six months post-operative

Complete healing was reached after removing sutures on the 30th postoperative day. At 6 months control no relapse was observed (fig.4).

## Discussion

To date, there is no therapeutic gold standard for ranula. In fact, several approaches have been proposed over the years with the aim of providing both therapeutic efficacy and minimal invasiveness, considering that the lesion occurs mainly in the pediatric age (3). In any case, the surgical techniques proposed up to 2007 did not achieve this aim; some procedures are decisive but very invasive with high risk of complications (4,6), while, on the other hand, other procedures are correlated with a high risk of recurrence (2,4,5,7). In 2007, modified micro-marsupialization was proposed with excellent results in terms of efficacy and very low recurrence rate^9^. The surgical technique is based on the following "rules": 1) greater number of sutures based on the size of the lesion, 2) shorter distance between the entry point and the exit point of the needle, 3) permanence of the suture for at least 30 days. These three crucial points allow the complete drainage of the mucus and, above all, the reduction of duct of Rivinus’s lumen portion by stimulating the epithelialization process (5,11). 

Indeed, the permanence of the sutures allows the progressive leakage of saliva through the sutures, the collapsing of the wall of the ranula, and its subsequent scarring that prevents its recurrence. So, modified micro-marsupia- lization is a minimal invasive and well tolerated procedure, simple and quick, with no post-operative complications and with a low risk of relapse. Furthermore, a 3-month follow-up is sufficient as the ranula tends to relapse within the first 50 post-operative days (9). Therefore, the better result with the better, less invasive and low-cost treatment is achieved, as performed in other ENT pathologies (5,14).

The only limit of this procedure is the impossibility of performing a biopsy of the lesion: therefore, its diagnosis remains clinical. In addition, the oral cavity is at high risk of infections and so it is essential to educate the patient on the management of oral hygiene and disinfection of the surgical wound in the post-operative period. Analyzing the literature, we identified only 15 cases of ranula treated with modified micro-marsupialization technique (9-13) (Table 1). Most of the patients were females (6 men: 8 women) with a mean age of 18.73 years (range 5 to 46 years). In our study, the patient was a 12-year-old male. No cases of recurrence and / or post-operative complications have been reported in the literature or in our study, demonstrating the high effectiveness and low invasiveness of modified micro-marsupialization.

**Table 1:** Review of literature about micro-marsupialization

**Case**	**Author (year)**	**Age**	**Gender**	**Duration of follow-up (months)**	**Outcome**
1	Sandrini et al. (2006)^9^	35	M	6	No recurrence
2		46	F	6	No recurrence
3		39	M	6	No recurrence
4	Amaral et al. (2012)^10^	16	F	18	No recurrence
5		12	M	13	No recurrence
6		8	M	12	No recurrence
7		15	F	12	No recurrence
8		9	F	10	No recurrence
9		30	F	9	No recurrence
10		16	M	7	No recurrence
11		15	M	7	No recurrence
12		5	F	6	No recurrence
13	Hegde et al. (2017)^11^	12	F	12	No recurrence
14	Matondkar et al. (2019)^12^	12	/	3	No recurrence
15	Silva et al. (2020)^13^	11	F	3	No recurrence
16	Present case	12	M	6	No recurrence

Recently, further modifications to this technique have been proposed: the use of resorbable sutures as an alternative to silk sutures in order to reduce the risk of infections and the "stich and stab method" which consists in only one suture applied on the roof of the ranula "drawing” four consecutives parallel strokes (10,13). Obviously more articles and patients are needed to confirm and validate the modified micro-marsupialization technique for treatment of ranula.

## Conclusion

Modified micro-marsupialization, introduced in 2007, is today the surgical technique of choice in the treatment of ranula. Due to its low invasiveness and its very low relapse rate, this surgical approach is strongly indicated and recommended, especially in pediatric patient, more often affected by this lesion. The poor case history found in the literature is probably an indication of the lack of knowledge of modified micro-marsupialization which, on the other hand, in our opinion, could be considered the gold standard, allowing to avoid “excessive” and ineffective treatments. In conclusion, this technique allows to achieve the better result with the better, less invasive and low-cost treatment.
